# Care delivery and compensation system changes: a case study of organizational readiness within a large dental care practice organization in the United States

**DOI:** 10.1186/s12903-017-0448-4

**Published:** 2017-12-20

**Authors:** Joana Cunha-Cruz, Peter Milgrom, Colleen E. Huebner, JoAnna Scott, Sharity Ludwig, Jeanne Dysert, Melissa Mitchell, Gary Allen, R. Mike Shirtcliff

**Affiliations:** 1 Northwest Center to Reduce Oral Health Disparities, Department of Oral Health Sciences, School of Dentistry, University of Washington, Box 357475, Seattle, WA 98195-7475 USA; 2 Northwest Center to Reduce Oral Health Disparities, Department of Oral Health Sciences, School of Dentistry, and Department of Health Services, School of Public Health University of Washington, Seattle, WA USA; 3 UMKC School of Dentistry, Kansas City, MO USA; 4 Advantage Dental Services LLC, Redmond, OR USA

**Keywords:** Organizational innovation, Quality improvement; healthcare reform, Dental care/manpower; patient care team, United States

## Abstract

**Background:**

Dental care delivery systems in the United States are consolidating and large practice organizations are becoming more common. At the same time, greater accountability for addressing disparities in access to care is being demanded when public funds are used to pay for care. As change occurs within these new practice structures, attempts to implement change in the delivery system may be hampered by failure to understand the organizational climate or fail to prepare employees to accommodate new goals or processes. Studies of organizational behavior within oral health care are sparse and have not addressed consolidation of current delivery systems. The objective of this case study was to assess organizational readiness for implementing change in a large dental care organization consisting of staff model clinics and affiliated dental practices and test associations of readiness with workforce characteristics and work environment.

**Methods:**

A dental care organization implemented a multifaceted quality improvement program, called PREDICT, in which community-based mobile and clinic-based dental services were integrated and the team compensated based in part on meeting performance targets. Dental care providers and supporting staff members (*N* = 181) were surveyed before program implementation and organizational readiness for implementing change (ORIC) was assessed by two 5-point scales: change commitment and efficacy.

**Results:**

Providers and staff demonstrated high organizational readiness for change. Median change commitment was 3.8 (Interquartile range [IQR]: 3.3-4.3) and change efficacy was 3.8 (IQR: 3.0-4.2). In the adjusted regression model, change commitment was associated with organizational climate, support for methods to arrest tooth decay and was inversely related to office chaos. Change efficacy was associated with organizational climate, support for the company’s mission and was inversely related to burnout. Each unit increase in the organizational climate scale predicted 0.45 and 0.8-unit increases in change commitment and change efficacy.

**Conclusions:**

The survey identified positive readiness for change and highlighted weaknesses that are important cautions for this organization and others initiating change. Future studies will examine how organizational readiness to change, workforce characteristics and work environment influenced successful implementation within this organization.

**Electronic supplementary material:**

The online version of this article (10.1186/s12903-017-0448-4) contains supplementary material, which is available to authorized users.

## Background

Increasingly, dental providers in the United States are employed in large dental group practices with 500 or more employees [1–3]. These care delivery systems, along with Federally Qualified Health Care Centers, have become part of the safety net for low-income enrolled children and pregnant women whose healthcare is paid for largely by the national and state governments [4]. Accompanying changes in delivery system complexity are initiatives to provide mobile community-based care, particularly in schools and preschools, to reduce barriers to care and mitigate costs. Government payments for dental services are moving from traditional fee-for-service to capitated or per member fixed payments with greater accountability for increasing the proportion of enrollees who receive care. Although low-income children enrolled in government insurance in the United States are entitled to dental care by law, utilization is low: in 2010, less than half of children had any dental care (46%) or preventive care (42%) [5].

“Organizational readiness to implement change” is a multi-faceted construct involving commitment to change and the structures and personnel necessary to enact or impede change [6]. Attempts to implement new systems in large organizations often fail because of lack of support from leaders to prepare staff members for changes [7]. Studies point to the importance of the organizational environment as associated with readiness [8–11]. Foundational work [7] and development of a theoretical framework [6] and measurement tools [12–15] have been published. For example, studies have examined readiness of physicians and nurses for changes in health information technology in primary care practices [16, 17] and in hospital care [18]. There has been no comparable research on dental care systems and practices as changes within these organizations are rapidly accelerating.

In this context, PREDICT – Population-centered Risk- and Evidence-based Dental Interprofessional Care Team – a quality improvement initiative to change the dental care delivery and compensation systems, was implemented by a large dental care organization serving low income children and pregnant women in rural Oregon. The objective of this case study was to assess organizational readiness for change before the implementation of system changes within the organization consisting of company owned clinics and contracted private practices. The study examined the association of organizational readiness with workforce characteristics and the work environment as perceived by dental care providers and supporting staff members. Based on previous research on organizational behavior [19], our primary hypothesis was that employees’ perceptions of organizational readiness to implement change would be associated with positive workforce characteristics such as their job satisfaction, perceptions of the organizational climate and support for the dental care model being implemented. Our secondary hypothesis was that employees within the central administration - outside of the clinics and practices - would be readier to implement change than would employees at the dental clinic or practice level. Our reasoning is that those closer to the leaders driving the change would be more involved, and more familiar, with the changes than those farther away from leaders. The project is part of the Robert Wood Johnson Foundation Finding Answers: Solving Disparities through Payment and Delivery System Reform program [20].

## Methods

### Context and setting

The setting of this case study is a quality improvement project (PREDICT [21]) conducted in 14 rural counties in Oregon, USA. The project is being implemented by a dental care organization, Advantage Dental Plans, LLC, Redmond, Oregon USA, The organization is a privately-owned for-profit company and one of the largest providers of dental care in Orgaon State for low income families insured by the United States’ joint federal-state program, Medicaid. In 2016, the organization had 42 group practices and contracted with approximately 200 affiliated smaller, largely rural primary care practices. Providers and staff members from the affiliated practices are not salaried employees of the organization and these practices receive from the organization either per capita monthly payments for patients assigned to them or discounted fee-for-service. Before this quality improvement project began, most clinical care was provided at dental clinics with limited outreach, and incentives paid only to providers were limited to only one performance measure based on access to care to pregnant women.

PREDICT involves changes at the system, community, provider and staff and patient levels [21]. The delivery system changes are intended to increase, substantially, community-based mobile care. At community settings, such as public schools and prenatal and early childhood nutrition and prevention agencies (e.g. Special Supplemental Nutrition Program for Women, Infants, and Children (WIC) and Head Start), Expanded Practice Dental Hygienists provide dental care needs assessment, risk-based preventive services, caries arresting, interim therapeutic restorative services and referrals to care to the company owned clinics or affiliated private practices. PREDICT follows a risk- and evidence-based prevention protocol, in which low risk children receive an assessment and fluoridated toothpaste once a year; moderate risk children receive the services provided to low risk children, as well as topical applications of 38% silver diamine fluoride (SDF) twice a year or 5% sodium fluoride varnish four times a year to the teeth; high risk children receive, in addition to the above, topical applications of 10% povidone iodine and 5% sodium fluoride varnish twice a year. Children with untreated active tooth decay receive 38% silver diamine fluoride and/or interim therapeutic restorations of glass ionomer cement to arrest the decay in specific teeth. The Expanded Practice Dental Hygienists who provide these services are supported by regional community liaisons responsible for establishing and maintaining the local partnerships needed to provide services in the community settings and by case managers who can follow-up with parents to obtain consent for community-based care and assist them with scheduling office appointments, as needed, for more complex care.

Changes in compensation are also part of PREDICT. Compensation changes are based, in part, on pay for performance and the performance metrics are designed to reduce disparities in access to care faced by the low-income patients. Provider and staff incentives are funded centrally from funds withheld on the payments to clinics. A broad range of employees are eligible to receive the incentive funds, including those working directly with clients at the community level, staff with the central administration (e.g., supervisors, case managers and information technology specialists) and clinic dental providers and staff (within the group and affiliated practices). The delivery system and compensation changes that comprise PREDICT are supported by an information technology system that facilitates ongoing performance feedback and quality improvement.

### Design

The study design was a cross-sectional survey conducted prior to implementation of PREDICT. The survey instrument and evaluation plan was submitted to the Institutional Review Board of the University of Washington for consideration. It was determined that this effort did not meet the definition of human subjects research.

### Sample selection and recruitment

The sample frame was all providers and staff in the group and affiliated private dental practices in the 14 counties and administrative staff at the company’s headquarters. Potential participants were invited to participate via email and the company’s internal newsletter. The company sent invitations to email addresses of all employees from staff model clinics and headquarters and sent invitations to one email address in the private dental care practices (either the dentist-owner or the practice’s generic email). Invitations to the private practices asked the email recipient to share the email with their employees. The invitations were repeated three times to encourage participation. Potential participants were informed that completion of the survey was voluntary, responses would be confidential and that no one within the company would review individual responses. Upon completion of the survey, respondents could provide their contact information (kept separate and unlinked from their responses) to enter a drawing for a tablet computer. Data was collected from July 15, 2015 through September 15, 2015.

### Procedures and instruments

Data collection was by a web-based questionnaire implemented by company information technology staff members using SurveyMonkey® (Palo Alto, CA). Usability and technical functionality of the questionnaire was tested in advance with company volunteers.

We developed a questionnaire comprised of 14 constructs; most item response options were 5- or 4-point Likert scales. Readiness for change was assessed by the Organizational Readiness for Implementing Change (ORIC) scales [14]. This validated 10-item questionnaire includes two scales that capture employees’ opinion that the people of the company are committed to the proposed changes (change commitment: Cronbach’s α=0.95) and the belief that the company can handle the adjustments needed for smooth and effective implementation (change efficacy: α=0.93). Response options for these items are 5-point Likert-scales that range from strongly disagree to strongly agree (1 to 5 points).

We developed specific items to assess opinions about the process and practice of dental care provided in the PREDICT model; for these, item response options used a 5-point Likert scale (from strongly agree to strongly disagree). Based on the results of a factor analysis, we created a 4-item scale representing support for the PREDICT model; the four items were respondent’s opinion about: 1) the company’s responsibility for obtaining parental consent for child’s dental care, 2) delivery of risk-based preventive care, 3) a priority focus on preventive care for children with greatest risk and 4) timely restorative and urgent care (α=0.79). Questions about employee’s agreement with the company’s mission statement and the use of methods for caries arrest or stabilization, such as silver diamine fluoride, were assessed separately.

The majority of constructs examined were adapted from the Minimizing Errors/Maximizing Outcomes (MEMO) questionnaire [19]. Organizational climate was measured with a single item about office or practice chaos and the organizational climate scale (α=0.95) that includes 5 subscales: workplace emphasis on quality (11 items, α=0.87), workplace cohesiveness (3 items, α=0.79), trust in the organization (5 items, α=0.86), workplace emphasis on information and communication (4 items, α=0.56) and leadership and governance alignment (8 items, α=0.87) [19]. Other workforce issues were assessed by a validated job stress scale (4-items, α=0.84) [22], a job satisfaction scale (5-items, α=0.81) [23], and single items on burnout [24] and likelihood to leave practice [19]. Data on demographics (i.e., respondent age, gender and race/ethnicity), job role, tenure, part-time or full-time employment and office or practice location were also collected. We categorized place of work as being “central” or “local.” Respondents’ with job responsibilities directly related to the company’s administration were considered “central”. We considered community liaisons and Extended Practice Dental Hygienists to be part of the “central” work group also because they were directly supervised by central administration staff. Providers and staff whose primary place of work was a dental practice were considered “local”. The survey questionnaire is available online as an Additional file 1.

### Statistical analysis

We estimated that a sample size of 51 participants would yield 80% power to detect a 1-point difference in ORIC mean scores between the two study groups (central vs. local), assuming an intra-county correlation (ICC) of 0.05 and standard deviation of 1.25. Frequencies and percentages for categorical responses and medians and interquartile ranges for continuous responses were calculated. If a respondent failed to complete more than 50% of the survey or if more than 30% of respondents failed to complete a specific question, that participant or question was excluded from the analysis. We calculated summary scores for the scales as the mean of the items. If more than 50% of items from a scale for a respondent was missing, the score was set to missing. If less than 50% of items were missing, we imputed the simple mean of the non-missing items for each respondent. Some item responses were reversed so that a higher summary score for the scale reflected a more positive attribute. We used linear regression models to investigate the association between organizational readiness for change and support for PREDICT with workforce characteristics and work environment. We tested for differences in readiness for implementing changes between respondents working at the central administration level and at the local dental practice level using linear regression models with an interaction term between central/local work group and workforce characteristics and work environment.

## Results

One hundred ninety-five questionnaires were received (28% of the estimated number of potential respondents). Fourteen participants had more than 50% of survey responses missing and were excluded from further analysis. No question had more than 30% of missing responses. The median time to complete the survey was 13 min. Of 181 participants included in the analysis, the majority were female (76%) and from non-Hispanic white race/ethnicity groups (78%). Approximately one-third of the respondents were in each of the following age categories: 18 - 32 years old, 33 - 44 years old and 45 or older. Most respondents worked for the company 1 to 5 years (41%), about a third for less than 1 year (27%) and the remaining 23% for 5 years or more. One third of the respondents worked for the company’s central administration, 54% worked locally in the 14 PREDICT evaluation counties and 13% worked locally in counties outside of the PREDICT evaluation counties or had missing practice/office location. Of the 54% who worked in the PREDICT evaluation counties: 40% were in pilot counties and 14% in comparison counties; 11% were dentists and 43% were clinic staff.

### Workforce characteristics and work environment

On a scale from 1 to 5, job satisfaction was high (median: 4.0), job stress and office chaos were moderate (median: 3), and burn out and likelihood to leave practice or office were low (median: 2).

**Fig. 1:**
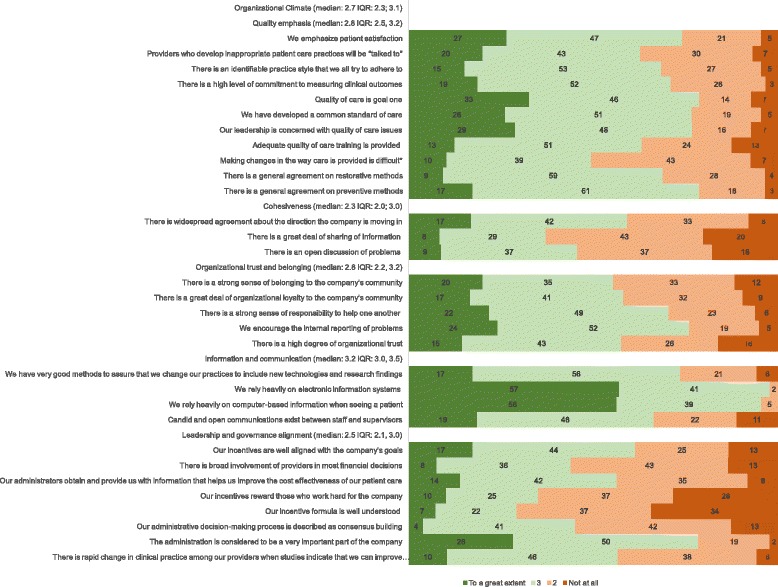
Organizational Climate (median IQR) from a dental care organization in 2015: Percentage of agreement with each statement of the scales on quality emphasis, cohesiveness, organizational trust and belonging, information and communication and leadership and governance alignment. (*N* = 181)

The overall assessment of organizational climate was moderate to high (2.7 on a 1 to 4 scale where 4 means best climate). The median scores for the subscales were very close to the median score for the scale ranging from 2.3 to 3.2 with the subscales of quality emphasis, organizational trust and information and communication above the median and subscales cohesiveness and leadership and governance alignment below the median. Items that scored negatively (a score of “not at all” or “2”) by 50% or more of respondents were: “There is a great deal of sharing of information” (63%) and “There is an open discussion of problems” (50%) from the cohesiveness subscale; and “Our incentives reward those who work hard for the company” (60%), “Our incentives is well understood” (71%) and “Our administrative decision-making process is described as consensus building” (55%) in the leadership and governance alignment subscale (Fig. 1).

### Support for the components of PREDICT and the company’s mission

**Fig. 2:**
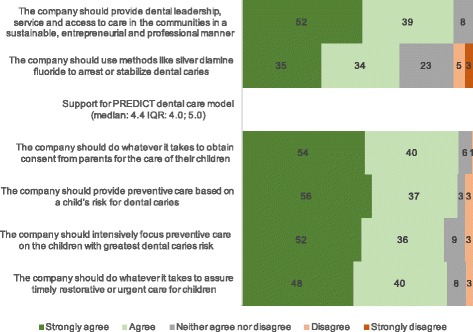
Percentage of agreement with each statement on the questions specifically related to the PREDICT dental care model from a dental care organization in 2015 (median IQR) (*N* = 181)

The overall median score on the 4-item scale that characterizes PREDICT’s approach to patient care (i.e., parental consent, risk-based care, preventive and timely care) was 4.4 (Interquartile range (IQR): 4, 5 on a 1 to 5 scale where 5 means greater agreement) (Fig. 2). There was strong alignment with the company mission to "provide dental leadership, service and access to care in the communities in a sustainable, entrepreneurial and professional manner." Sixty-nine percent of the respondents strongly agreed or agreed that the company should use methods to arrest or stabilize dental caries such as silver diamine fluoride.

### Innovation outcomes: Organizational readiness to implement change

**Fig. 3:**
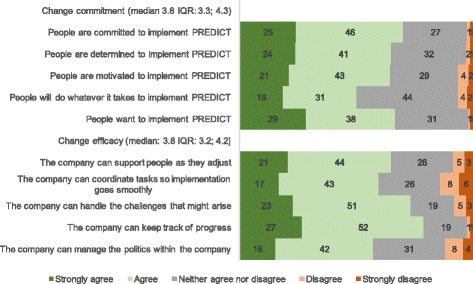
Organizational Readiness to Implement Change related to the PREDICT dental care model from a dental care organization in 2015 (median and IQR): Percentage of agreement with each statement of the scales on change commitment and change efficacy (N = 181)

Organizational readiness for implementing change was high: median change commitment was 3.8 (IQR: 3.3, 4.3) and median change efficacy was 3.8 (IQR: 3.0, 4.2 on a 1 to 5 scale where 5 means maximum readiness). More than 50% of respondents strongly agreed or agreed with the statements from the two organizational readiness to change scales (Fig. 3).

### How are support for PREDICT and organizational readiness to implement change related to workforce characteristics?

In the unadjusted analysis, support for PREDICT was associated with change commitment (b: 0.22; 95%CI: 0.10, 0.33), change efficacy (b: 0.17; 95%CI: 0.06, 0.27), organizational climate (b: 0.0.33; 95%CI: 0.15, 0.50) and job satisfaction (b: 0.12; 95%CI: 0.00, 0.24), but they lost statistical significance after adjustment for demographics and other workforce characteristics. Before and after adjustment, support for PREDICT was positively associated with support for the company’s mission (adjusted b: 0.41; 95%CI: 0.25, 0.56) and for caries arrest treatments (adjusted b: 0.12; 95%CI: 0.02, 0.22). There were no differences in support for PREDICT components and organization’s mission between the respondents working at the central administration level and at the local dental practice level. Other workforce characteristics were not associated with support for PREDICT.

In the unadjusted regression analysis, both change commitment and change efficacy scales were positively associated with greater support for PREDICT, for the company’s mission and for caries arrest treatments, organizational climate, job satisfaction and being a central staff member as opposed to being a dentist or staff at the clinics. Change commitment and change efficacy were inversely associated with higher burnout, office/practice chaos and likelihood to leave practice/office. Change efficacy was also inversely associated with job stress (Table 1).

**Table 1:** Association between implementation scales and employees’ characteristics and opinions from a dental care organization in 2015: Linear regression coefficients and 95% confidence intervals (*n* = 141)

	Change Commitment Scale				Change Efficacy Scale			
	Crude b [95% CI]		Adjusted b^$^ [95%CI]		Crude b [95% CI]		Adjusted b^$^ [95%CI]	
Support for dental care model	0.43***	[0.20,0.65]	0.09	[−0.16,0.33]	0.37**	[0.13,0.61]	−0.03	[−0.22,0.17]
Support for company’s mission	0.47***	[0.27,0.67]	0.15	[−0.09,0.38]	0.61***	[0.41,0.81]	0.20*	[0.02,0.39]
Support for caries arrest treatments	0.31***	[0.19,0.44]	0.16*	[0.02,0.30]	0.37***	[0.24,0.50]	0.06	[−0.05,0.17]
Organizational climate	0.79***	[0.57,1.01]	0.45**	[0.16,0.75]	1.20***	[1.02,1.39]	0.80***	[0.57,1.04]
Job satisfaction scale	0.39***	[0.24,0.55]	0.09	[−0.13,0.32]	0.63***	[0.49,0.78]	0.08	[−0.10,0.26]
Job stress scale	−0.08	[−0.24,0.08]	0.18	[−0.02,0.38]	−0.32***	[−0.48,-0.16]	0.08	[−0.08,0.24]
Burn out	−0.14*	[−0.27,-0.01]	0.06	[−0.12,0.25]	−0.42***	[−0.54,-0.29]	−0.19*	[−0.33,-0.04]
Office chaos	−0.22*	[−0.40,-0.05]	−0.19*	[−0.38,-0.01]	−0.30**	[−0.48,-0.11]	0.01	[−0.14,0.15]
Likelihood to leave practice	−0.19***	[−0.29,-0.08]	−0.06	[−0.18,0.06]	−0.33***	[−0.43,-0.23]	−0.08	[−0.17,0.02]
Central staff	0.33*	[0.06,0.61]	0.09	[−0.17,0.34]	0.39**	[0.10,0.68]	0.04	[−0.17,0.24]

*p < 0.05, **p < 0.01, ***p < 0.001

^$^The adjusted models contain all variables listed in the table and age, gender, race/ethnicity

When the results were adjusted for demographics and all other variables in the regression model, change commitment remained associated with support for caries arrest and inversely related to office chaos; change efficacy remained associated with support for the company’s mission and inversely related with burn out. Both change commitment and change efficacy remained positively associated with organizational climate. For each one unit increase in the organizational climate scale, 0.45 and 0.8 unit increases in change commitment and change efficacy scales were predicted (Table 1). Interaction terms between being in the central administration versus being sited in a clinic or affiliated practice and workforce characteristics and work environment were not statistically significant.

## Discussion

During a period of dental care system change, we conducted a survey of employees in a large dental care organization in Oregon State, USA to assess organizational readiness to change. The results of this case study demonstrated a high level of readiness to implement change, both in change commitment and change efficacy. Consistent with the literature outside of dentistry, perception of a positive organizational climate was a significant predictor of organizational readiness [8–11]. These findings highlight the influence of employees’ perceptions of quality of care, organizational cohesiveness, trust, belonging, leadership and communication on their willingness to enact changes in the workplace to address oral health disparities. The company’s mission states a goal of providing care for everyone in the community. Employees’ agreement with the mission statement was strongly associated with their support for PREDICT’s goals to improve access to care through community-based services and in their belief in the company’s competence to manage the changes necessary to implement PREDICT.

Employees’ support for using methods to arrest tooth decay in the field predicted higher change commitment – the shared resolve to implement changes – and higher support for the PREDICT model. It is worth noting that one delivery system change implemented in PREDICT is topically applied 38% silver diamine fluoride to prevent and arrest decay, a product that was cleared by the United States Food and Drug Administration only in 2014 [25]. The results of our survey suggest that providers and staff members who are open to new technologies are also more accepting of changes in healthcare settings.

High-quality, timely and accurate communication about changes is associated with readiness [10, 12, 18]. In this study, we found that communication and decision-making processes could be improved. Within general positive opinions, many employees disagreed that there was open discussion of problems and that decisions were made without a process of consensus building. The results also showed that the incentive program was not well understood. Staff and providers work throughout the State of Oregon and communication between them and the central administration implementation team can be improved. Inadequate communication about the nature and expectations for change can frustrate staff and providers thus dampening their initial enthusiasm and motivation for new initiatives to succeed. A clear communication plan with the involvement of central staff, leaders, local staff and providers is needed to maximize the uptake and sustainability of the PREDICT model beyond the initial implementation stage.

Interestingly, the hypothesis of greater readiness to change among employees of the central administration versus those at the local dental practice level were not confirmed. We attribute this finding to how the changes were planned and implemented. The planning/implementation group was made up largely of senior employees. Many of the centralized personnel who would be part of the community care teams were not hired or assigned to these jobs until after the program planning was well along. Thus, our assumption that they would have greater involvement, beginning with the inception of the project, was not supported by the data in this study.

### Limitations

Participation in the survey was voluntary and we did not have email contact information from all potential non-dentist participants who were employed within primary care practices. We relied on the dental provider from the primary care clinics to encourage participation by staff members. Respondents from central administration and local practice were similar in terms of sex, race/ethnicity, age and job role. Nevertheless, response rates were higher for central administration staff than the clinics and practices, and the overall low response rate limits generalization.

## Conclusions

This case study is an important first step in developing a literature on organizational change within the evolving dental care system in the United States. The initial survey demonstrated readiness for implementing changes but also highlighted areas of weakness that are important cautions for others initiating organizational change. Future studies within this organization will provide insights on how readiness to implement changes, workforce characteristics and work environment impacted the success of implementation and intended outcomes of the PREDICT model. Similar studies are needed in other settings to address the applicability of the larger organizational change literature, as well as these case specific findings, to the larger dental care delivery system.

## Additional file

Additional file 1: PREDICT_Survey_Questionnaire_2015-093.pdf - Survey Questionnaire. The file contains the questionnaire used in this study. (PDF 114 kb)

## Abbreviations

ICC: Intra-county correlation
IQR: Interquartile range
ORIC: Organizational readiness for implementing change
PREDICT: Population- risk- and evidence-based dental interprofessional care team
SDF: Silver diamine fluoride
