# Supplementary material for: Care delivery and compensation system changes: a case study of organizational readiness within a large dental care practice organization in the United States
Source: BMC Oral Health. 2017 Dec 20;17:157. doi: 10.1186/s12903-017-0448-4 (PMC5738825; doi:10.1186/s12903-017-0448-4)
Supplement: Additional file 1: — PREDICT_Survey_Questionnaire_2015-093.pdf - Survey Questionnaire. The file contains the questionnaire used in this study. (PDF 114 kb) [file 12903_2017_448_MOESM1_ESM.pdf]

Advantage
PREDICT
Provider and Staff Survey

CONFIDENTIAL

University of Washington
Funded by the Robert Wood Johnson Foundation

Advantage Dental Services is changing how care is delivered and how PCDs and employees are incentivized using a new model called PREDICT – Population- Risk- and Evidence-based Dental Interprofessional Care Team. The goal is to dramatically increase the number of Advantage members who receive dental care.

PREDICT is being implemented by Advantage central administration in 7 counties and the effect of the changes will be compared to services provided in 7 comparison counties.

PREDICT focuses on gaining consent from parents and providing risk-based preventive care in community settings and timely restorative and urgent care in dental offices for children, adolescents, pregnant and post-partum women.

Because the changes impact many parts of the company, everybody's opinion is important to assure the success of Advantage's initiative.

1. Whether or not you are directly involved, please circle the number that best describes how much you agree or disagree with each statement in relation to PREDICT implementation.

**PREDICT implementation**	Strongly disagree	Disagree	Neither agree nor disagree	Agree	Strongly agree
**People who are part of the Advantage community …**					
a. … are committed to implement PREDICT.	1	2	3	4	5
b. … are determined to implement PREDICT.	1	2	3	4	5
c. … are motivated to implement PREDICT.	1	2	3	4	5
d. … will do whatever it takes to implement PREDICT.	1	2	3	4	5
e. … want to implement PREDICT.	1	2	3	4	5
**Advantage can …**					
f. … communicate effectively.	1	2	3	4	5
g. … support people as they adjust to PREDICT.	1	2	3	4	5
h. … coordinate tasks so implementation goes smoothly.	1	2	3	4	5
i. … handle the challenges that might arise.	1	2	3	4	5
j. … keep track of progress.	1	2	3	4	5
l. … manage the politics within the company.	1	2	3	4	5
**Advantage should …**					
m. … do what is needed to obtain consent from parents for the care of their children.	1	2	3	4	5
n. … expect parents to arrange care for their children.	1	2	3	4	5
o. … provide preventive care based on a child's risk for dental caries.	1	2	3	4	5
p. … intensively focus preventive care on the children with greatest dental caries risk.	1	2	3	4	5
q. … provide less preventive care to children with low dental caries risk.	1	2	3	4	5
r. … use silver diamine fluoride to arrest or stabilize dental caries.	1	2	3	4	5
s. … do whatever it takes to assure timely restorative or urgent care for children.	1	2	3	4	5
t. … leave it to parents to be responsible for timely restorative or urgent care for children.	1	2	3	4	5
u. … provide dental leadership, service and access to care in the communities in a sustainable, entrepreneurial and professional manner.	1	2	3	4	5

2. Which number best describes the atmosphere in your workplace?

- ☐ 1. Calm
- ☐ 2
- ☐ 3 Busy, but reasonable
- ☐ 4
- ☐ 5 Hectic, chaotic

3. Using your own definition of "burnout", please check *one* of the options below*:*

☐ 1 I enjoy my work. I have no symptoms of burnout.

☐ 2 Occasionally I am under stress, and I don't always have as much energy as I once did, but I don't feel burned out.

☐ 3 I am definitely burning out and have one or more symptoms of burnout, such as physical and emotional exhaustion.

☐ 4 The symptoms of burnout that I'm experiencing won't go away. I think about frustrations at work a lot.

☐ 5 I feel completely burned out and often wonder if I can go on. I am at the point where I may need some changes or may need to seek some sort of help.

**4. Please assess how well each statement describes *your own work situation* overall .**	Strongly disagree	Disagree	Neither agree nor disagree	Agree	Strongly agree
a. I find my work personally rewarding.	1	2	3	4	5
b. I am pleased with my work.	1	2	3	4	5
c. I am satisfied to be in my workplace.	1	2	3	4	5
d. My work situation is a major source of frustration.	1	2	3	4	5
e. My work in this workplace has not met my expectations.	1	2	3	4	5
f. I feel a great deal of stress because of my job.	1	2	3	4	5
g. Very few stressful things happen to me at work.	1	2	3	4	5
h. My job is extremely stressful.	1	2	3	4	5
i. I almost never feel stressed at work.	1	2	3	4	5
j. I feel competent to work with patients and families that have different cultural background.	1	2	3	4	5
l. My experience working with patients and families with different cultural backgrounds has been positive.	1	2	3	4	5

**5. What is the likelihood that you will leave your work within TWO YEARS?**	None	Slight	Moderate	Likely	Definitely
	1	2	3	4	5

**6. To what degree do the following statements reflect the conditions in your workplace within Advantage or affiliated with Advantage?**	Not at all			To a great extent
a. There is widespread agreement about the direction the company is moving in.	1	2	3	4
b. There is a great deal of sharing of information.	1	2	3	4
c. There is an open discussion of problems.	1	2	3	4
d. There is a strong sense of belonging to the Advantage community.	1	2	3	4
e. There is a great deal of organizational loyalty to the Advantage community.	1	2	3	4
f. There is a strong sense of responsibility to help one another.	1	2	3	4
g. We encourage the internal reporting of problems.	1	2	3	4
h. There is a high degree of organizational trust of Advantage.	1	2	3	4
i. Our incentives are well aligned with Advantage's goals.	1	2	3	4
j. There is broad involvement of providers in most financial decisions.	1	2	3	4
k. Our administrators obtain and provide us with information that helps us improve the cost effectiveness of our patient care.	1	2	3	4
l. Our incentives reward those who work hard for Advantage.	1	2	3	4

m. Our incentive formula is well understood.	1	2	3	4
n. Our administrative decision-making process is described as consensus building.	1	2	3	4
o. The administration is considered to be a very important part of Advantage.	1	2	3	4
p. There is rapid change in clinical practice among our providers when studies indicate that we can improve quality/reduce costs.	1	2	3	4
q. We emphasize patient satisfaction.	1	2	3	4
r. Providers who develop inappropriate patient care practices will be "talked to".	1	2	3	4
s. There is an identifiable practice style that we all try to adhere to.	1	2	3	4
t. There is a high level of commitment to measuring clinical outcomes.	1	2	3	4
u. Quality of care is goal one.	1	2	3	4
v. We have developed a common standard of care.	1	2	3	4
w. Our leadership is concerned with quality of care issues.	1	2	3	4
x. Adequate quality of care training is provided.	1	2	3	4
y. Making changes in the way care is provided is difficult.	1	2	3	4
z. There is a general agreement on restorative methods.	1	2	3	4
aa. There is a general agreement on preventive methods.	1	2	3	4
bb. We have very good methods to assure that we change our practices to include new technologies and research findings.	1	2	3	4
cc. We rely heavily on electronic information systems.	1	2	3	4
dd. We rely heavily on computer-based information when seeing a patient.	1	2	3	4
ee. Candid and open communications exist between staff and supervisors.	1	2	3	4
ff. There is a great deal of respect to patients and families from all cultural backgrounds.	1	2	3	4

A LITTLE MORE ABOUT YOU

1. What is your age? ______

2. What is your sex?

☐ Male

☐ Female

3. What is your ethnicity? (please choose one)

☐ Hispanic/Latino

☐ Not Hispanic/Latino

4. What is your race? (check all that apply)

a. White (European, Middle Eastern, other) ☐

b. Black or African American ☐

c. American Indian or Alaska Native ☐

d. Native Hawaiian or Pacific Islander ☐

e. Asian ☐

f. Other: ______________________________ ☐

5. What is your job title?

____

5B. Which of the following best describes your job activities?

Dentist	Front Desk	Customer Service
EPP Dental Hygienist	Sterilization Tech	Admin
EPP DH - Community	Regional Manager	Marketing
Dental Hygienist	Case Manager	Management
Dental Assistant	IT Staff	Corporate Executive
Office Manager	Financial	

6. Do you work full-time or part-time at your primary workplace?

☐ Full-time

☐ Part-time (less than 40 hours per week)

7. How long have you worked as part of Advantage community?

_____ years _____ months

8. Are you an owner (shareholder or partner) of Advantage Dental?

☐ Yes

☐ No – **skip to #15 [IF NOT THE PCD PRACTICE OWNER, GO TO #15].**

9. How many shares do you own?

____

10. How many child and adolescent (< 21 years old) OHP members are assigned to your practice? ____

11. How many adult OHP members are assigned to your practice? ____

12. Please describe the payment arrangement for services received by patients of your practice:

a. Self-pay or uninsured	____%
b. Capitated care	____%
c. Fee-for-service/discounted fee-for-service	____%
d. Other	____%

13. In your practice, what percent of your insured patients are:

a. Oregon Health Plan (OHP)	____%
b. Commercial Insurance including ODS	____%
c. Other	____%

14. Please estimate the percent of your patients in the following categories:

	Patients
Age	
a. Infants (0-< 24 months)	____%
b. Preschool children (2 – 5 years)	____%
c. Children (6 - 12 years)	____%
d. Youth (13 – 20 years)	____%
e. Adult (21 – 64 years)	____%
f. Elderly (65 and older)	____%

	Patients
g. Female	____%
h. Pregnant women	____%
i. Speak little or no English	____%
j. Have complex or numerous dental problems	____%
k. Have complex or numerous medical problems	____%
l. Have complex or numerous psycho-social problems	____%
m. Are generally frustrating to deal with	____%
n. Have alcohol or other substance abuse disorders	____%

15. Where is your office located? (check only one)

Coos
Deschutes
Douglas
Jackson
Josephine
Klamath
Morrow
Wasco
Crook
Curry
Grant
Jefferson
Lake
Lincoln
Umatilla
Advantage's Central Administration
Other county
Specify ________________________

16. Which is the primary county you serve? (check only one)

Coos
Deschutes
Douglas
Jackson
Josephine
Klamath
Morrow
Wasco
Crook
Curry
Grant
Jefferson
Lake
Lincoln
Umatilla
Advantage's Central Administration
Other county
Specify ________________________

17. Do you serve any other counties? (check all that apply)

☐ Coos
☐ Deschutes
☐ Douglas
☐ Jackson
☐ Josephine
☐ Klamath
☐ Morrow
☐ Wasco
☐ Crook
☐ Curry
☐ Grant
☐ Jefferson
☐ Lake
☐ Lincoln
☐ Umatilla
☐ Advantage's Central Administration
☐ Other county
Specify ________________________

Thank you for taking the time to take our survey.
